# Comparison on Quality Performance of Human Hair Types with Herbal Oils (Grape Seed/Safflower Seed/Rosehip) by Analysis Techniques

**DOI:** 10.1021/acsomega.2c06550

**Published:** 2023-02-22

**Authors:** Ecem Demir, Nil Acaralı

**Affiliations:** Department of Chemical Engineering, Yildiz Technical University, Davutpasa Campus, Esenler-Istanbul 34220, Turkey

## Abstract

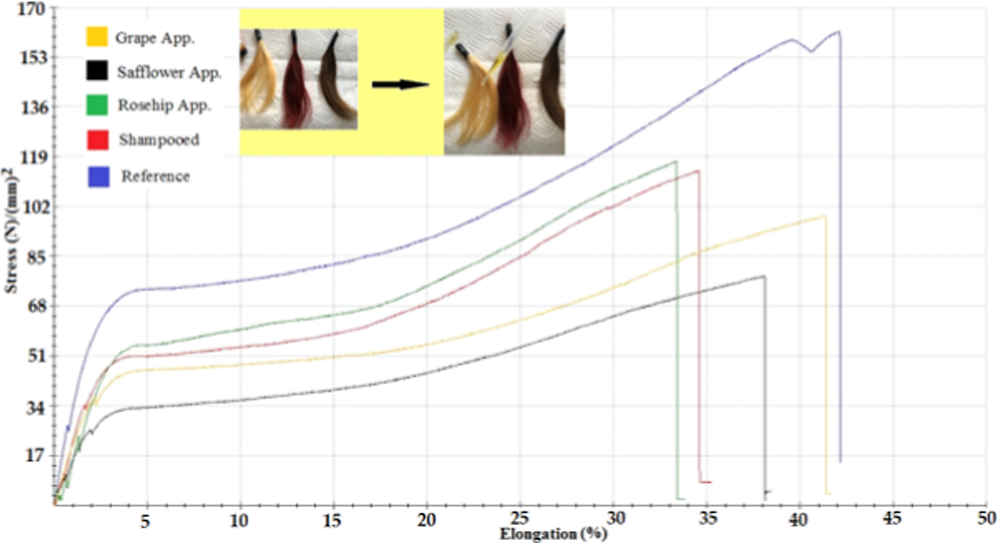

Hair is exposed to harmful factors such as sunlight, pollution, cosmetic applications, and cleaning every day. With lost moisture, the hair is worn out, loses shine, and exhibits color changes in the case of dyed hair. In this study, the effects of herbal oils on hair were investigated by comparing the properties with measurements. Three different types of hair were used: natural (unprocessed), damaged, and dyed hair. After washing hair with a base shampoo, herbal oils were applied, and brightness, color changes, elasticity, and breaking points were examined. Safflower seed oil, grape seed oil, and rosehip oil were applied to the samples. It was tried to regain the properties that have decreased as a result of shampoo application in the hair with the applied oils. The highest gloss was observed with grape seed oil, and according to color change calculations, the best result was seen with safflower seed oil. Tensile-strain testing was performed for all samples, and rosehip oil gave the best results. The changes in hair fractures were examined with a scanning electron microscope, and grape seed oil was the best for all hair types. When all analyses were evaluated, the best performing herbal oil was grape seed oil. All analysis results showed that herbal oils can be used in the cosmetics industry with different applications.

## 1 Introduction

Hair is a filament-like extension of the epidermis found in mammals and is made up of a proteinaceous substance called large-step keratin.^1^ For the owner of the hair, it can act as camouflage and to attract sexual partners as a mechanical sensor.^2^ Regardless of the hair type, hair always grows from a hair follicle, which is an extension of the epidermis. Lanugo hair is the type of hair that sheds right after birth. Vellus hair is very thin and covers the whole body. Terminal hair, on the other hand, is the longer and coarser hair type that covers the scalp. The body is covered with small hair fibers, while the scalp, armpits, and the genital area are covered with stronger terminal hair fibers. From the point of view of a cosmetic scientist, the most important hair type is the terminal type of hair.^3^ The elastic properties of hair are generally defined by examining the relationship between stress, applied force, and deformation caused by the treatments/techniques. In practice, the tensile and elastic properties of a single hair strand could be measured by stretching the hair at a constant rate and recording the stress/strain curve. Determination of the tensile properties of single hair fibers was standardized and carried out on a tensile tester with elongation to break. If the tests were done properly, the results were reproducible and allowed statements about hair quality and the extent of hair damage. Playing an important role was a combination of statistical analysis as well as complementary methods. No two hair fibers were the same, and therefore, measurements were associated with a high variability. The most likely explanation for the existence of this type of melanin structure was that they were synthesized from tyrosine found in keratin. The second type of melanin pigment found in hair is a type of pheomelanin, formerly known as trichodermins. They are complex aromatic structures but much lighter in color than eumelanin and often impart a yellow-red color to the hair. The final color of the hair would depend on the ratio of eumelanin and pheomelanin. The chromophores produced by both are easily destroyed by reducing agents such as hydrogen peroxide, resulting in bleaching and discoloration. Colorimetric methods allowed not only the correct determination of the color values in the hair curls but also the calculated formulation of the desired shades. A prerequisite was the creation of calibration curves for different colors. This method could be used to determine the degree of damage to the hair, as colors were absorbed differently by damaged hair, for example, by bleaching, cold wave, abrasion, or splitting. Hair shine is a very important feature for the cosmetic industry because high shine (gloss) is desired by consumers. A hair fiber in good condition is very shiny due to the very flat, amorphous cuticle platelets on the outside of the hair shaft. This smooth surface reflects light normally, with little scattering, and produces a characteristically high degree of gloss. Psychophysical methods for assessing gloss were quite satisfactory, but errors occurred due to the difficulty of visually assessing gloss, the dependence of gloss on the alignment of the curls, and the fact that gripping hair would alter the reflective properties of the hair surface.^3,4^ Most cosmetic hair treatments are topical, and therefore, the surface properties of the fiber are of great importance to the cosmetic chemist. The cuticle is the outermost layer that protects the internal structures of the hair; it has the most contact with the outer environment. Therefore, as a fiber is pushed out of the follicle, it has very smooth and unbroken scaling edges as shown. The layered structure is uniformly packed. However, as the fibers grow, the hair is broken, the cuticles are opened, the hair gets worn, and the cuticles appear to be lifted from the hair surface. This type of damage is usually caused by weather conditions and mechanical damage such as combing and brushing. This phenomenon is more pronounced in long strands of hair.^5,6^ Damage to the cuticle on the hair surface also affects the tensile properties.^7^ Hair is not of vital importance to humans. However, hair is an important element of people’s body image because it is important psychologically and socially as a part of one’s identity.^8^ People use a variety of hair care products according to their needs. These are shampoos, conditioners, styling products, straighteners, and dyes.^9^ In recent years, consumers have begun to be more concerned about their appearance in terms of aesthetics and health. They expect to get the most out of their cosmetic products. It is important for the manufacturer to see the effectiveness of the raw materials in the cosmetic product.^10^ Today, there is an increasing consumer demand for personal care products including ingredients that are natural and organic. According to the demand, growth has occurred in the natural and organic personal care cosmetic products sector.^11^ The biggest factors in the growth of the ingredients market are that they should be healthier, organic, and ecological. Consumers are now examining the ingredient composition of personal care products and demanding products containing natural extracts and ethical and certified organic ingredients.^12^ In the literature, botanical ingredients used in personal care products include purified plant components.^13^ The researchers discussed the effects of acids, bases, and various types of reagents, such as oxidizing agents and reducing agents, through a quantitative method to determine how much the various bond types in the hair fibers contribute to the amount of mechanical strength in their study of the mechanical properties and structure of the hair. Using Fick’s second diffusion law, the diffusion coefficient of the hydroxyl ions in the hair was calculated.^14^ The researchers investigated the effectiveness of botanical extracts on hair, and the effects of three botanical actives on hair were measured. As a result of this study, it was decided that botanical active substances based on peptides and proteins etc. could be utilized to protect and repair hair fibers.^15^ Leite et al. (2018) investigated the photoprotective effects of cosmetic products including botanical extracts, vitamins, and UV filters. In the study, the researchers developed and evaluated the effectiveness of a multifunctional hair care cream containing tea, grape, and acai berry extracts. In conclusion, the multi-functional conditioner formulation demonstrated the various benefits of a single product effective in preventing UV damage and hair damage.^16^ Cloet et al. (2020) tried to understand the mechanical properties of curly hair and the strength of the hair strand in their study. The relationship between the geometric and mechanical profiles of hair strands was studied, focusing specifically on curly hair samples. The main result of the study was that the tensile strength of hair fibers consists of two components.^17^ In the work of Thieulin et al. (2019), the effects of cosmetic applications on the morphology and sensory properties of a single strand of human hair were investigated. In daily life, human hair is involved in various mechanical and chemical processes such as straightening, combing, drying, and washing, and these processes can cause cracks in the cuticle of the hair. The results showed that all treatments alter the morphology of the hair by damaging the hair cuticle.^18^ Yu et al. (2016) examined the structural and mechanical behavior of hair. As a result of the study, the contribution of elastic and plastic deformations of α-keratin fibers was evaluated and correlated with structural changes.^19^ Richena and Rezende (2016) investigated the morphological deterioration of the human hair cuticle due to simulated sunlight irradiation and washing. The results obtained from the study showed that the endocuticle and cell membrane complex were cuticle structures that deteriorated more with sunlight irradiation. The most severe effect was seen in samples where irradiation and washing were combined. These samples resulted in a more pronounced cuticle extraction.^20^ Fernandez et al. (2012) investigated the effects of antioxidants on human hair. The UV components of sunlight damaged the hair and caused deterioration in the hair fiber structure. Lipidic peroxidation of UV-induced protein degradation was decreased for some processed fibers. This point was more evident in fibers processed using artichoke extract; however, rice extract was shown to better preserve the shine and color of hair fibers.^21^ Richena and Rezende (2015) conducted a study called the effect of photodamage on the outermost cuticle layer of human hair. The results presented that after irradiation with a mercury lamp, small spherical shaped nodules appeared on the cuticle surface and the size of these nodules increased with increasing irradiation time.^22^ Guthrie et al. (1995) carried out a study to investigate the factors affecting hair coloring with dyes from the Arianor series. Significant changes in the surface structure have been found to occur in relatively light treatments of human hair.^23^ Zülli et al. (2001) reported that antioxidants from grape seeds protected the hair against reactive oxygen species. It was concluded that the application of antioxidants based on procyanidins-derived seeds could protect hair against reactive oxygen species.^24^ The studies in the literature showed that various seed oil types could be evaluated in many industries such as cosmetics, pharmaceutical, and agro industries.^25−27^ The literature also showed that safflower, rosehip, and grape seed oils were utilized as agents inducing hair growth to improve the wave effect, to decrease the damage to the hair, to accelerate hair regeneration, and to promote hair follicle cells.^28−30^

The use of vegetable oils in personal cosmetics is widespread nowadays; thus, in this study, the effects of the raw materials on hair were examined, and it is hoped to make a great contribution to the cosmetics industry and the literature. The aim of this study was to observe the effects on human hair in detail of using the oils of plants such as safflower, rosehip, and grapes grown in Turkey.

## 2 Materials and Methods

The study was conducted on three hair types: natural, worn, and dyed hair. Five samples were prepared for each of these hair types.

### 2.1 Materials

Hair dye, botanic brand hair bleaching powder, and volume oxidation cream were provided by a cosmetics firm. Natural dark brown hair samples were taken from Imhair (Italy). A base shampoo formula was prepared for shampoo application to the hair samples (Table 1). In addition to the given formulation, an oxidizing agent concentration was used at a maximum of 0.05% (w/w) for wearing out the hair. Also, the oxidizing agent concentration was utilized at a maximum of 1% (w/w) for dyed hair.

**Table 1:** Base Shampoo Formula Prepared for Shampoo Application to Hair Samples

ingredients	% (w/w)
water	71.50
acrylates/alkyl acrylate crosspolymer and propylene glycol	7.00
sodium laureth sulfate and cocamidopropyl betaine	19.00
phenoxyethanol (and) methylparaben (and) ethylparaben	0.50
sodium hydroxide and sodium chloride	2.00

The herbal oils used (safflower seed oil, grape seed oil, and rosehip oil) were bought from the market. The major fatty acid of safflower seed oil was linoleic acid, which accounted for 70% in the oil. The rosehip seed oil contained polyunsaturated fatty acids, linoleic acid (54%), linolenic acid (19%), phytosterols, and β-sitosterol (82%). The grape seed oils contained stearic acid (6%), palmitic acid (9%), oleic acid (15%), and linoleic acid (70%).

### 2.2 Methods

The weight and density of the samples were selected as the standard for color analysis. Temperature and humidity are crucial for stress–strain, color, and the other tests. All hair samples selected for the applied analysis were approximately 20 cm long and 30 g in weight. In this way, stability was ensured between samples. To determine whether the properties of the hair samples were improved or not, scanning electron microscopy (SEM) analyses were carried out in order to determine the cuticle status as well as tests for color, gloss, elongation, stretching etc. were conducted. In particular, Δ*E* values, which were a significant criterion for determining the distance between colors, were calculated, and the results were evaluated.

The shampoos were evaluated in terms of stability, considering the physicochemical specifications, force, stress, elongation, rheological properties, dirt dispersion level, foaming ability, and foam stability. All shampoos gave good results with a percentage of solids, foam formation with a stable structure, and a viscous nature. According to all test results, the shampoo samples were within the specified range with good wetting ability.

#### 2.2.1 Preparing Worn Hair

**Figure 1:**
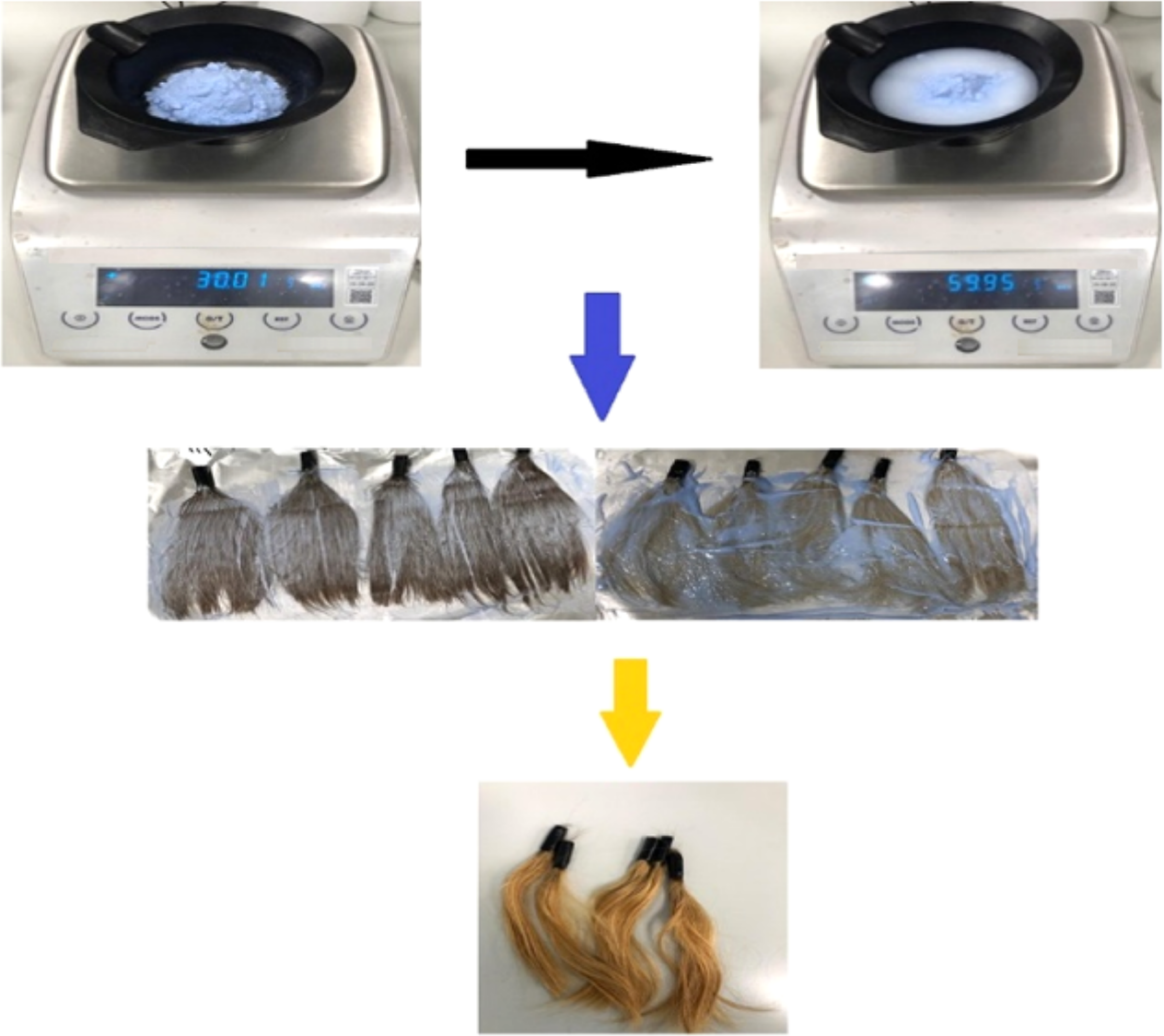
Images of hair bleaching powder weighing, oxidation cream weighing, bleaching of worn hair, and worn hair samples.

The hair color used in the study contained aqua, ammonia, cetylic-stearylic alcohol, and *para*-phenylenediamine. The *para*-phenylenediamine content was not more than 2.0% after dilution. Hair bleaching powder and volume oxidation cream were weighed at a ratio of 1:1. During the hair coloring preparation stage, first the oxidant and then the hair color were put into a mixer bowl at the determined rates. They were mixed until they were well homogenized. The oxidant in the specified ratio was used for one tube of hair color. The mixture was applied to five samples and left for 1 h. After 1 h, the hair was cleaned with water and dried. The same mixture was prepared again and applied to the dried hair and left for 40 min. The hair was purified from the bleaching mixture with water and dried (Figure 1).

#### 2.2.2 Preparing Dyed Hair

**Figure 2:**
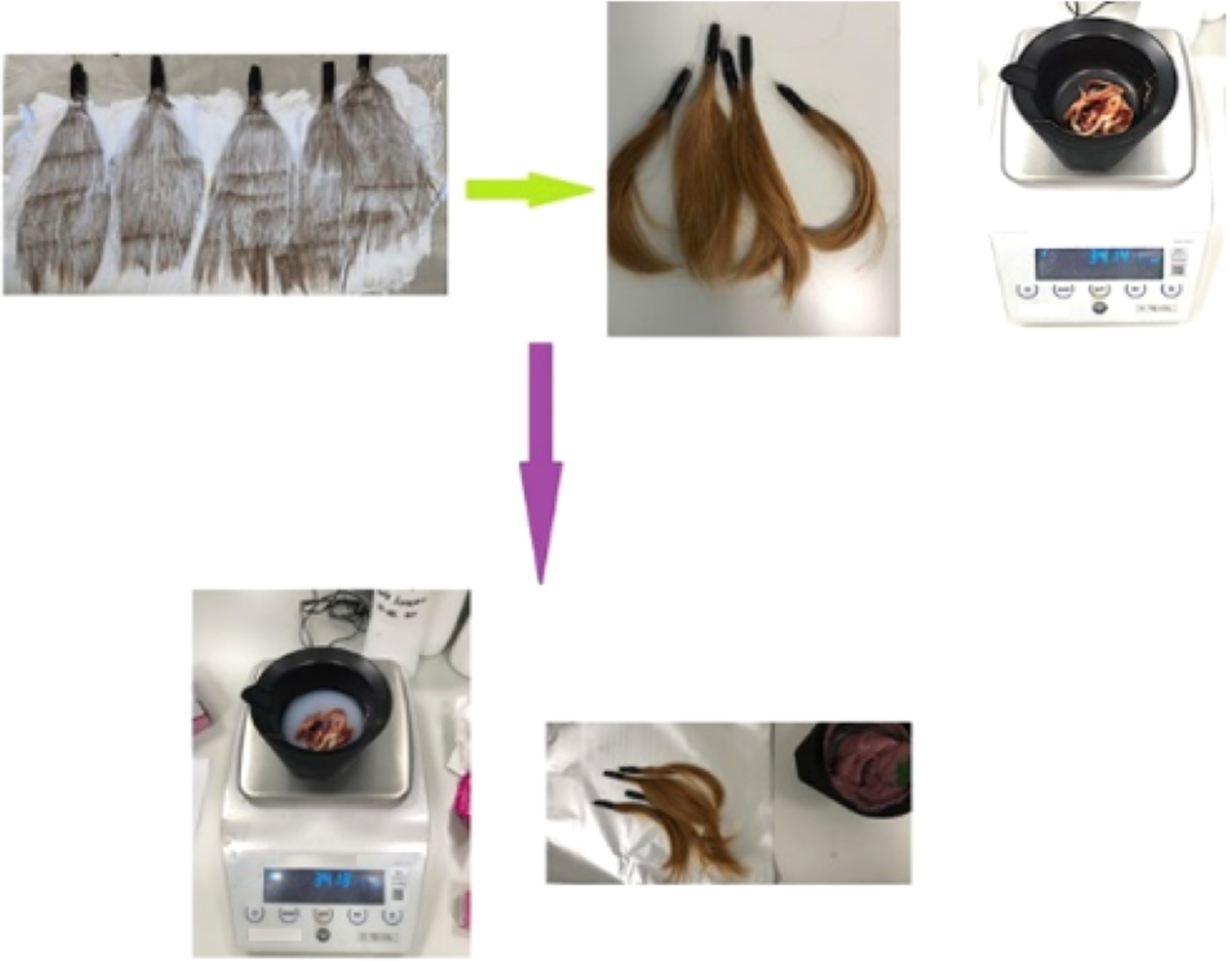
Hair samples dyed with bleach, bleached hair dyed, hair dye weighting, oxidation cream weighting, and dyeing of hair samples.

A botanic brand powder hair lightener and a volume oxidation cream were weighed and mixed with a 1:1 ratio for five of the tied hair samples. The mixture was applied to the five samples and left for 30 min. After 30 min, the hair was washed with water and dried (Figure 6). Hair dye and volumes of oxidation cream were mixed in a 1:1 ratio. The dye prepared was applied to the dried hair and left for 30 min (Figure 2). The hair was washed, freed from the dye, and dried.

#### 2.2.3 Preparing Natural Hair

**Figure 3:**
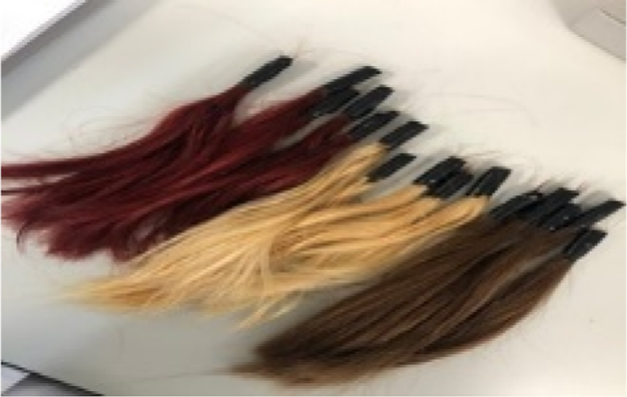
Prepared hair samples (left to right: dyed-worn-natural).

Natural hair was hair that had not undergone any processing. 5 of the hair samples by dividing into 15 parts were separated as natural hair samples. When all procedures were finished, the hair samples were ready for trials (Figure 3).

#### 2.2.4 Applying Oils to Hair

**Figure 4:**
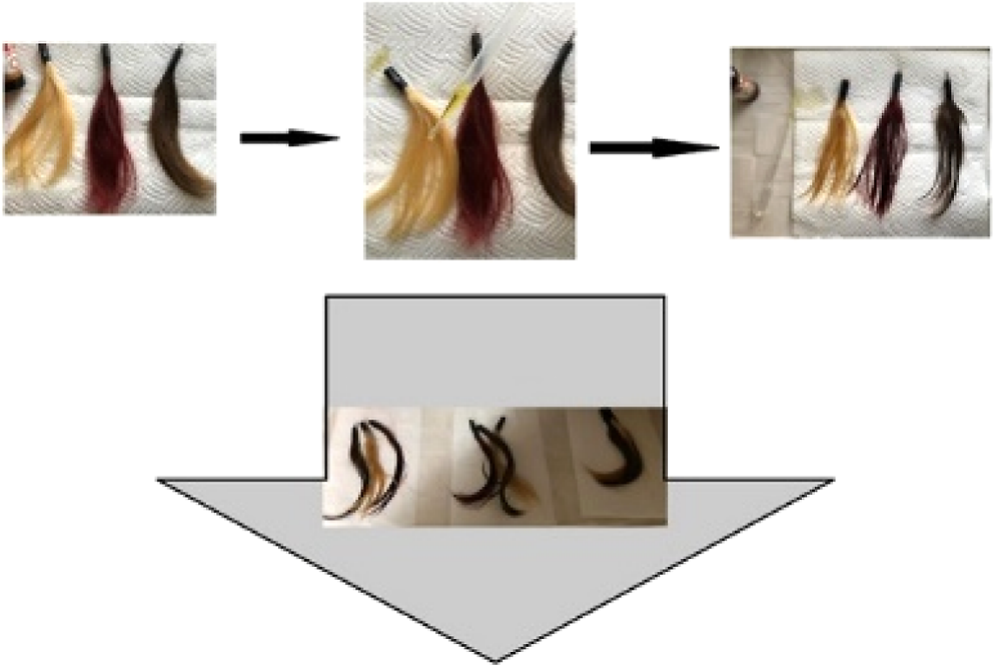
Hair samples for applied oil, oil application, and hair samples after oil application.

One sample from each of the different types of hair samples was taken and set aside as the reference hair sample. One of them was set aside for shampoo application only. Safflower seed oil was applied to one, rosehip oil to one, and grapeseed oil to one of the remaining three hair samples from each hair type. Oils (0.5 mL) were applied to the hair with the help of a pipette. The oils were left on the hair for 12 h. While determining the optimum ratio for the oil applied to the hair, trials were carried out in the range of 0.1–1.0 mL. As a result of the tests and analyses such as color, stretching, elongation, morphological appearance, etc., no change was observed in the properties of the hair samples after a certain value. For this reason, all test and analysis results were compared in detail, and the optimum amount to be applied to the hair was determined as 0.5 mL. This rate corresponded to a rate of 1.5% (v/w) over the determined amount of hair. In practice, the minimum time for all applications to the hair was 12 h. In addition, because of the preliminary trials performed between 6 and 24 h, there was no significant change in the analysis and test results after 12 h. Therefore, the optimum time was determined as 12 h. After 12 h, the hair was washed with a base shampoo and oiled again. This washing, drying, and oiling process was repeated 10 times. After the 10th lubrication, the hair was washed again with a base shampoo, dried, and ready for tests. Some images taken while applying oil to hair samples are given in Figure 4. In this experiment, safflower seed oil, grape seed oil, and rosehip oil were used comparatively.

#### 2.2.5 Stress–Strain Test

The tension-strain test was applied to the hair strands selected from each of samples that had been subjected to oil applications. Stress and strain testing was performed in a standard atmosphere (20 °C and 65% RH). The test device used in study could break or stop, depending on the distance, and its sensitivity was ±0.5%. Also, the speed setting ranged from 2 to 500 mm/min. The hair strands were attached to a special equipment in the device for compressing the hair strands. Accordingly, the tensions (N/mm^2^) and elongation (%) of the hair strands were recorded. Samples of the same hair type were shown on the same graph.

#### 2.2.6 Gloss Measurement

Gloss measurements of the hair samples were made with standard angles of 20, 60, and 85°. The weight and size of the hair samples taken for gloss and color measurements were approximately 100 grams and 20 cm, respectively. The hair samples were combed and placed on a black surface. The measurement was made by placing the device on the hair sample without losing the roughness of the samples and without distorting their shape. Measurements were made under standard atmospheric conditions (20 °C and 65% relative humidity). Since the placement of the hair sample and the device had a very significant effect on the brightness, the measurements were repeated three times at three angles. Then, the average of these three values was taken for each angle.

#### 2.2.7 Color Measurement

The samples were placed in the glass eye of the device, and reading was taken. In a color measurement, the lightness or darkness of the color was measured on the axis L* on a scale of 0 (black) to 100 (white). On axis a*, the value of the color between −100 (green) and +100 (red) was measured. The axis b* showed values between −100 (blue) and +100 (yellow), which was the criterion for hair color. It was known that each unit on L*, a*, and b* axes formed the smallest color difference that the human eye could.^21,31^ Using the a*, b* color diagram and measured values, the color of the samples could be determined. To reduce the margin of error caused by the misposition of the sample, three readings were made and averaged for each sample. Total color loss (Δ*E*) was figured out by evaluating the changes in the L*, a*, and b* readings on the hair samples with a spectrophotometer color meter.

 was a criterion to find the ’distance’ between different colors. In calculations, the color change (Δ*E*) of each hair sample relative to the reference hair of its own hair type was calculated using eq 1. Also, Δ*E* values were compared for natural, dyed, and worn hair types. Δ*L*, Δ*a*, and Δ*b* were calculated from the difference of *L*_2_ – *L*_1_, *a*_2_ – *a*_1_, and *b*_2_ – *b*_1_, respectively.
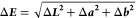
1

### 2.3 Scanning Electron Microscopy

Depending on the procedures performed, a SEM analysis study was carried out with hair samples to find possible changes in the physical surface morphology of hair strands.

## 3 Results and Discussion

The purpose of using vegetable oils in this study was to contribute to the improvement of color, shine, stretching, elongation, and breaking properties of hair samples subjected to different processes. As a result, when all analysis results were evaluated, it was seen that vegetable oils had a positive effect on the specified properties.

### 3.1 Stress–Strain Test

**Figure 5:**
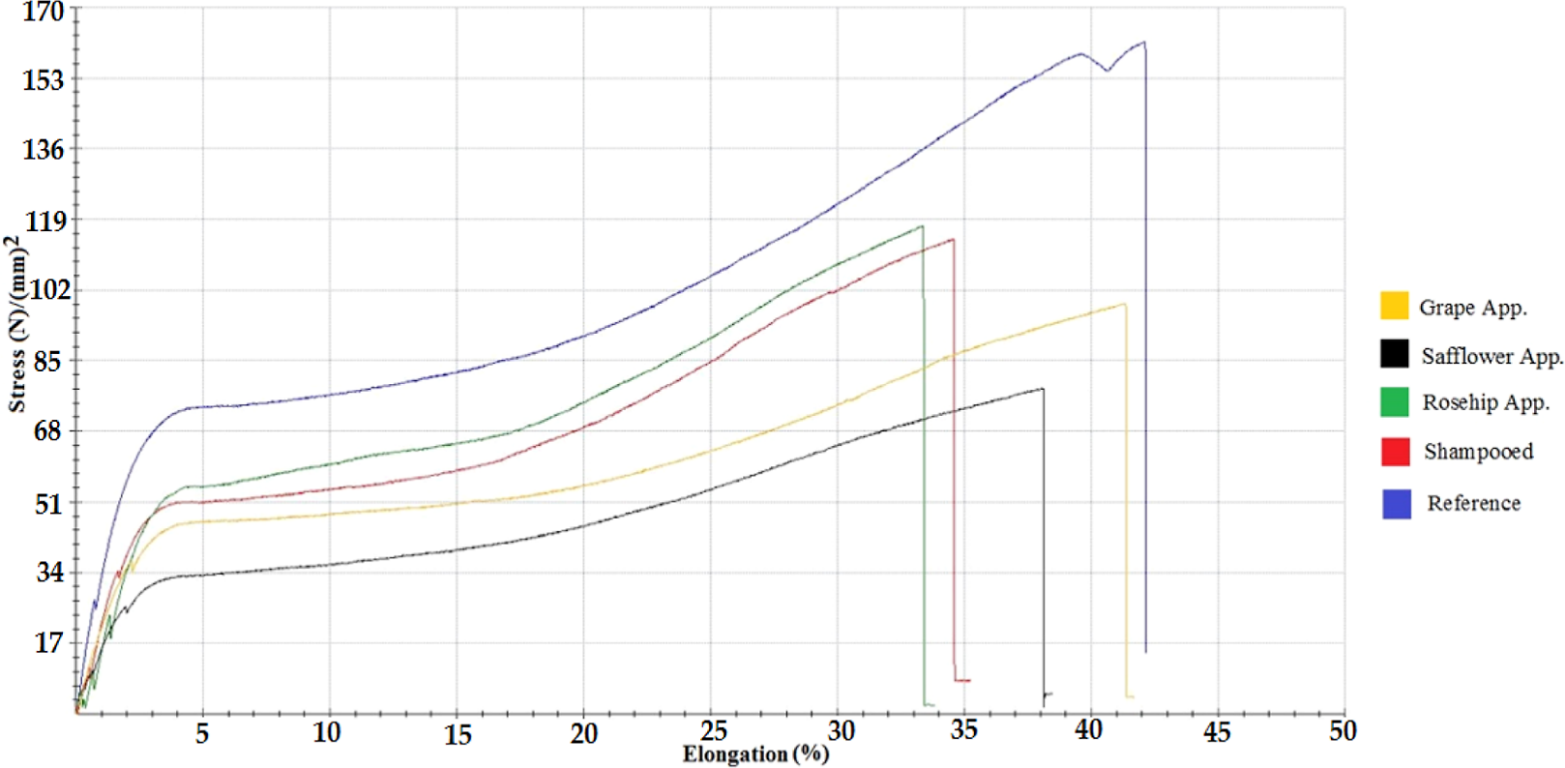
Stress–strain test graph for natural hair.

A random strand of hair was taken from the samples and tightly attached to both ends of the equipment and compressed. The device strength and length were reset, and the pull was started and was automatically graphed. In these graphs, the rupture point, stretching, and stretching rates of the hair were read. The stress–strain test graph for natural hair samples is given in Figure 5 and the table of measured values is given in Table 2. The lower the value of the refractive force, the more damage occurred in the cortex.^10^ When the result was evaluated by comparing the point of rupture, hair with the highest force value could be interpreted as the strongest hair. When evaluated using this criterion, the order from the hair sample with the highest voltage rupture point to the hair sample that broke at the lowest voltage was as follows: reference natural hair, natural hair with rosehip oil applied, shampooed natural hair, natural hair with grape seed oil applied, and natural hair with safflower seed oil applied.

**Table 2:** Stress–Strain Test Values for Natural Hair/Worn Hair/Dyed Hair

sample	max. force (*F*_max_) (N)	*F*_max_ elongation(Δ*l*_max_) (mm)	*F*_max_ elongation(ε_max_) %	max stress (σ_z_B) (N)/(mm)^2^	flow limit (σ_s_) (N)/(mm)^2^	rupture force (*F*_K_) (N)	rupture elongation (Δ*l*_K_) (mm)	rupture elongation (ε_K_) %
reference	1.277	42.06	42.06	162.5927	25.8468	0.116	42.154	42.154
	0.725	46.465	46.465	92.3099	15.1516	0.043	47.236	47.236
	0.88	42.692	42.692	112.0451	10.4406	0.025	43.263	43.263
shampooed	0.903	34.553	34.553	114.9735	7.1301	0.063	35.271	35.271
	0.241	2.013	2.013	30.6851	24.7008	0.03	2.616	2.616
	1.131	35.507	35.507	144.0034	21.5177	0	35.544	35.544
rosehip app	0.927	33.334	33.334	118.0293	4.8383	0.016	33.845	33.845
	0.608	29.628	29.628	77.413	5.8569	0.035	30.116	30.116
	0.789	35.707	35.707	100.4586	18.462	0.058	62.144	62.144
safflower app	0.619	38.04	38.04	78.8135	6.1115	0.038	38.447	38.447
	0.831	35.505	35.505	105.8062	2.0372	0.078	36.39	36.39
	0.337	4.605	4.605	42.9082	16.8068	0.003	27.099	27.099
grape seed app	0.781	41.305	41.305	99.44	36.4147	0.032	41.688	41.688
	0.776	37.314	37.314	98.8034	8.1487	0.044	37.647	37.647
	0.933	43.267	43.267	118.7932	25.2101	0.039	44.419	44.419
average (*x*)	0.9	37.86	37.86	114.77	16.07	0.05	38.28	38.28
	0.64	30.18	30.18	81	11.18	0.05	30.8	30.8
	0.81	32.36	32.36	103.64	18.49	0.02	42.49	42.49
Std. deviation(s)	0.2429	3.9039	3.9039	30.9255	14.2718	0.0392	3.7207	3.7207
	0.2358	16.8681	16.8681	30.0195	8.9401	0.0193	16.9047	16.9047
	0.2946	15.9465	15.9465	37.5098	5.5216	0.0251	12.9974	12.9974
CV %	26.9889	10.3114	10.3114	26.9456	88.8102	78.4	9.7197	9.7197
	36.8438	55.8917	55.8917	37.0611	79.9651	38.6	54.8854	54.8854
	36.3704	49.2784	49.2784	36.1924	29.8626	125.5	30.5893	30.5893

**Figure 6:**
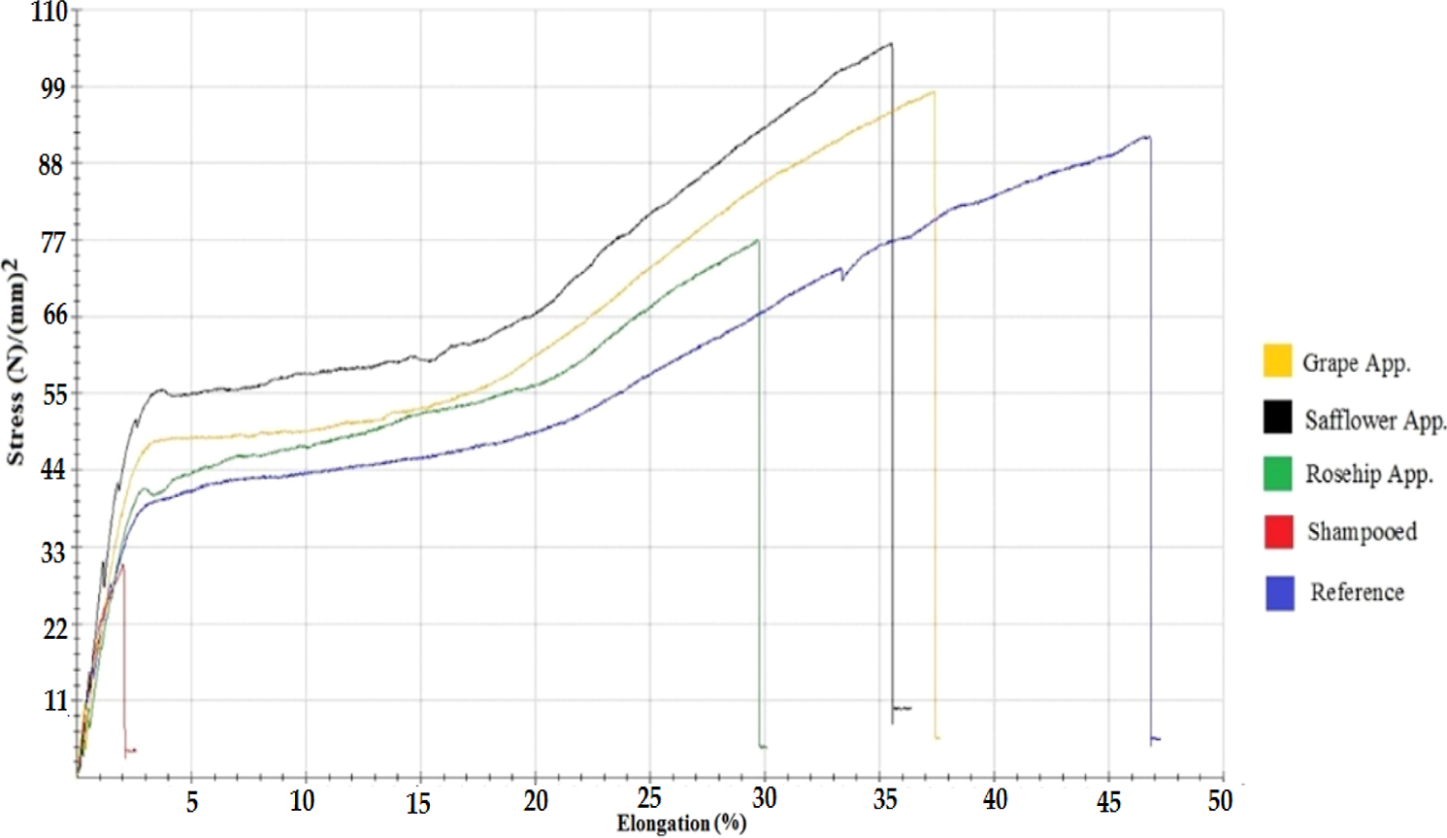
Stress–strain test graph for worn hair.

**Figure 7:**
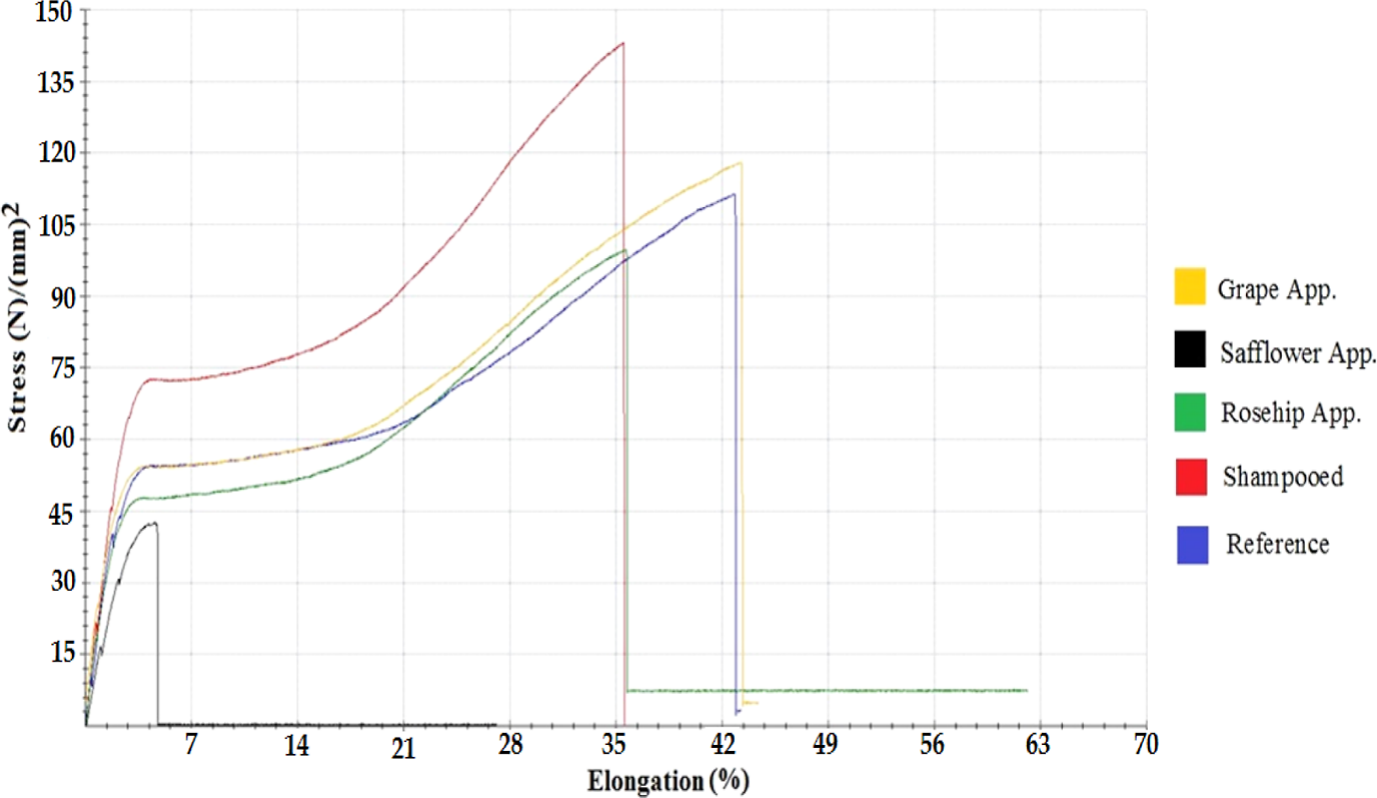
Stress–strain graph for dyed hair.

The stress–strain test graph for worn hair samples is given in Figure 6. The order of the hair samples, which had the breaking point at the highest voltage, based on the breaking point of the hair to the hair sample that broke at the lowest voltage was as follows: worn hair with safflower seed oil applied, worn hair with grape seed oil applied, worn hair with rosehip oil applied, reference worn hair, and shampooed reference hair. Accordingly, herbal oils performed well in the field of breaking point. It was seen that the use of herbal oils strengthens the hair. The stress–strain test graph for the dyed hair samples is given in Figure 7.

The ranking based on the breaking point of the hair was in the order from the hair sample with the break point at the highest voltage to the hair sample that broke at the lowest voltage: shampooed dyed hair, dyed hair with grape seed oil applied, reference dyed hair, dyed hair with rosehip oil applied. The dyed hair applied with safflower seed oil could not be included in this ranking because it broke in the elasticity area between 0 and 5%.

### 3.2 Gloss Test

Brightness measurements were made for 20, 60, and 85° with the device. The hair was placed on a black matte floor. The reading part of the device was placed on the hair so that it was extremely close to the hair. To minimize the error rate caused by the placement of the hair, three measurements were made for each sample, and the averages of these values were taken (Table 3).

**Table 3:** Gloss Measurements for Natural Hair/Worn Hair/Dyed Hair

	gloss measurements		
sample	20°	60°	85°
reference hair	0.2/0.2/0.2	0.77/2.27/0.6	0.93/0.83/0.93
shampooed hair	0.2/0.2/0.2	0.73/2.7/0.97	0.9/1.13/1.63
hair with rosehip oil applied	0.2/0.2/0.2	0.6/2.8/0.8	0.93/1.17/1.4
hair with safflower seed oil applied	0.2/0.2/0.2	1/2.27/0.87	1.6/0.93/1.3
hair with grape seed oil applied	0.2/0.2/0.2	0.9/3.13/1	1.5/1.03/1.3

In hair samples with safflower seed oil and grape seed oil applied, the brightness values increased considerably in both measurement grades. However, the best brightness values between the two were observed in the natural hair sample with safflower seed oil applied.

Accordingly, the brightness values of the treated hair samples were higher than the brightness value of the reference hair sample. The highest values at 60 °C were read in worn hair samples applied with grape seed oil, worn hair applied with rosehip oil, shampooed hair, and safflower seed oil applied.

At 85 °C, the highest value was obtained for the worn hair sample with rosehip oil applied. Then, shampooed worn hair, worn hair with grape seed oil applied, and hair with safflower seed oil applied were analyzed. Accordingly, the brightness values of the treated hair samples were higher than the brightness value of the reference hair sample.

The highest values at 60 °C were seen for dyed hair with grape seed oil applied, shampooed hair, dyed hair with safflower seed oil applied, and dyed hair with rosehip oil applied. The highest value at 85 °C was read for the shampooed hair sample.

### 3.3 Color Measurement

Color measurements of the samples were performed with a spectrophotometer. The samples were placed in the glass eye of the device and read. To reduce the margin of error caused by the misposition of the sample, three readings were made for each sample and their averages were taken (Table 4).

**Table 4:** Color Measurements for Natural Hair/Worn Hair/Dyed Hair

	color measurements		
sample	L	A	b
reference hair	23.73/47.89/20.68	4.18/3.9/11.02	6.227/19.05/3.26
shampooed hair	23.81/46.79/20.53	4.01/3.99/10.71	5.74/17.61/3.32
hair with rosehip oil applied	24.35/48.49/21.69	4.21/4.17/10.99	6.39/18.20/3.49
hair with safflower seed oil applied	24.16/46.67/21.80	3.98/4.85/11.44	6.01/18.52/3.97
hair with grape seed oil applied	23.44/48.18/22.35	3.95/4.26/11.82	5.45/18.38/4.30

In the experimental studies for natural hair, the color change seen in the shampoo applied hair sample was 0.525 compared to 0.648 for the hair sample applied with rosehip oil, 0.525 for the hair sample applied with safflower seed oil, and 0.858 for the hair sample applied with grape seed oil. Apart from the shampoo, it was safflower seed oil that caused the least discoloration in natural hair samples. In experimental studies for dyed hair, the color change seen in the shampoo applied hair sample was 0.355 compared to 1.034 for the hair sample applied with rosehip oil, 1.394 for the hair sample applied with safflower seed oil, and 2.120 for the hair sample applied with grape seed oil. Apart from the shampoo, rosehip oil caused less color change than other herbal oils. In the experimental studies for worn hair, the color change seen in the shampoo applied hair sample was 1.812 compared to 1.074 for the hair sample applied with rosehip oil, 1.637 for the hair sample applied with safflower seed oil, and 0.815 for the hair sample applied with grape seed oil. Among the worn hair samples, the least discoloration occurred in the sample where grape seed oil was applied.

#### 3.3.1 Color Change Calculations

The equations to be utilized for color change calculations are given as eqs 2–5. Δ*E* denotes the color change.
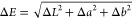
2
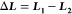
3
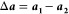
4
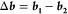
5

According to the calculations, color changes are given in the table. Accordingly, the color change (Δ*E*) of each hair type according to the reference hair in its own hair type were computed by using eqs 2–5. The calculated values are shown in Table 5. It was observed that the Δ*E* results ranged between 0.52 and showed results close to those for the reference hair.

**Table 5:** Color Changes of Samples

	Δ*E*		
sample	natural hair	dyed hair	worn hair
shampooed hair	0.525	0.352	1.812
hair with rosehip oil applied	0.648	1.034	1.074
hair with safflower seed oil applied	0.525	1.394	1.637
hair with grape seed oil applied	0.858	2.120	0.815

### 3.4 SEM Analysis

**Figure 8:**
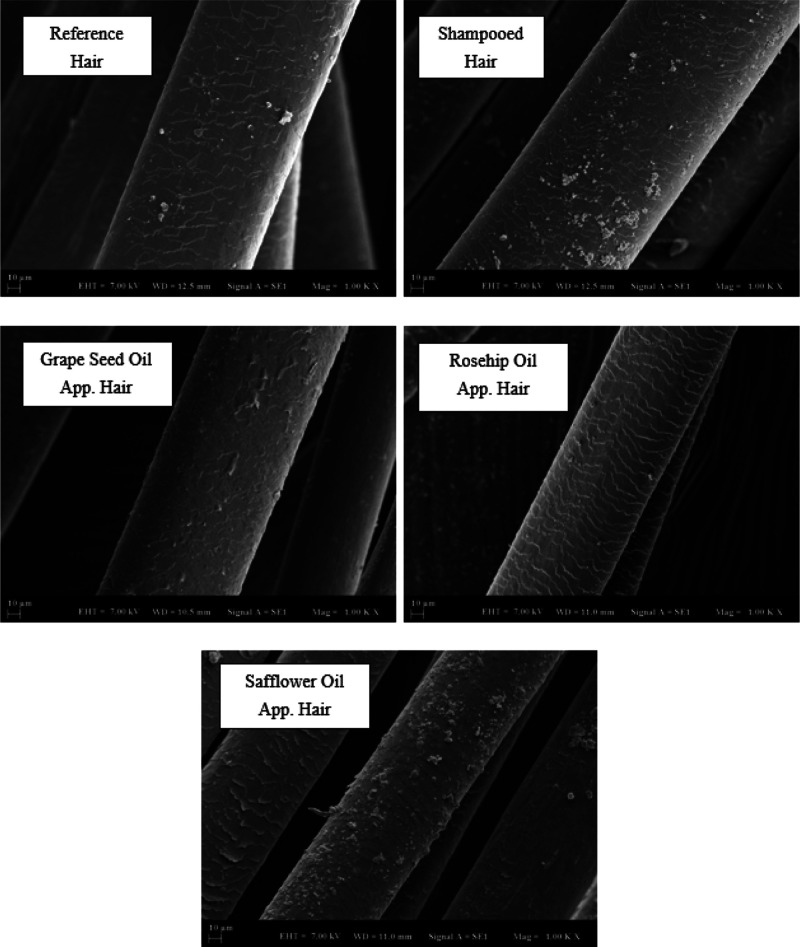
SEM analysis for natural hair (×1000).

SEM showed detailed, magnified images of hair by scanning its surface to provide a high-resolution image. In the results, the microscope was operated at 7.00 kV. The images in the range of WD = 11 ± 1 mm were selected from all images. Analytical conditions for SEM images were set at 10 micrometer and 1000x magnification. In the analysis method, hair samples were placed in the device, with a coating of gold, under vacuum to prevent glare and reflection. A comparison was made from Grade 0 to Grade 5 in the evaluation of the hair samples. This comparison was graded according to the deterioration of the cuticle structure. Compared to the literature, the best grade (Grade 0) in which the cuticle was intact was determined by the SEM analysis results in the morphological images of the hair follicles.^32^ In Figure 8, SEM analysis images are shown for natural hair samples. SEM images of control bleaching treatment revealed damaged cuticle edges. The changes occurring on the hair surface were observed due to the procedures done on the natural hair sample. The reference hair had a smooth appearance as it did not go through any procedure. Grape oil reduced hair wear, while safflower oil was found to wear out more. There was no change with the application of rosehip oil.

**Figure 9:**
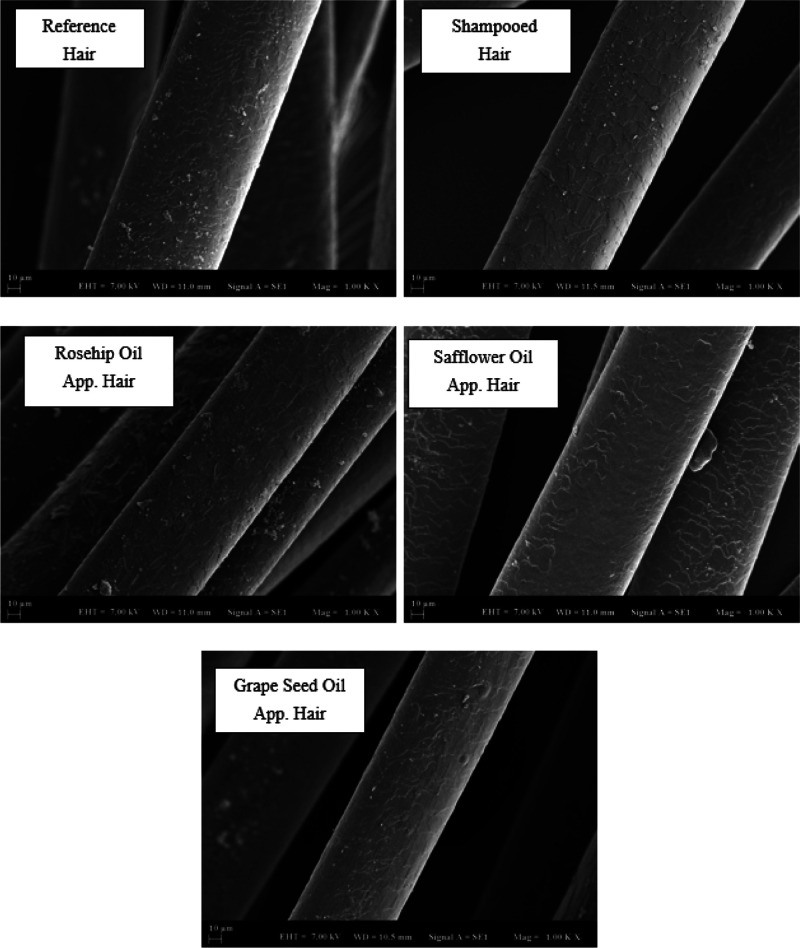
SEM analysis for worn hair (×1000).

In Figure 9, SEM analysis images are given for worn hair samples. As shown in the figure, morphological changes on hair surface can be seen because of the procedures done on the worn hair sample. It was seen that there were improvements on the surface of the hair compared to the reference hair. The best repair was seen in hair strands with safflower and grape oil application.

**Figure 10:**
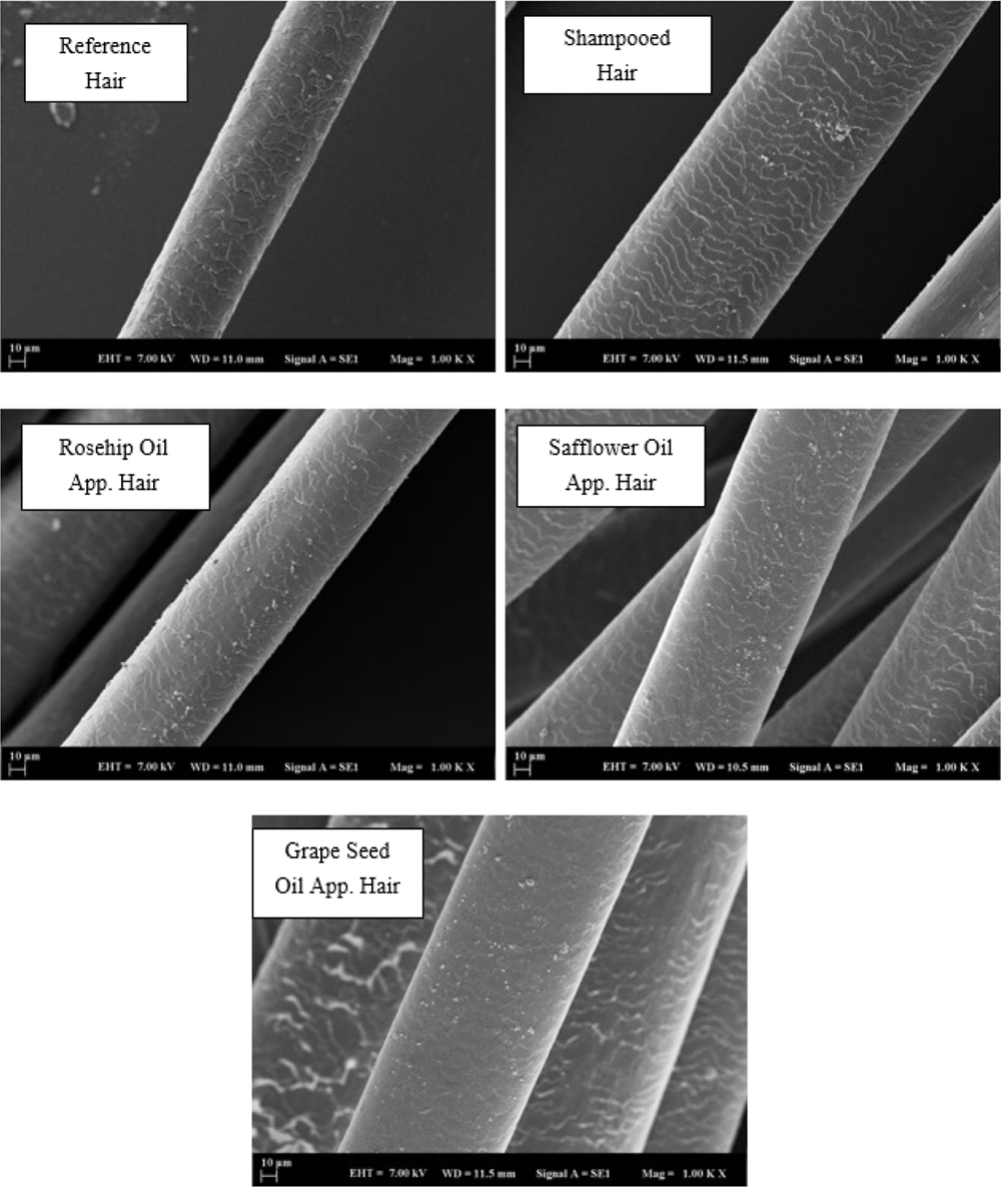
SEM analysis for dyed hair (×1000).

In Figure 10, SEM analysis images for dyed hair samples are exhibited. The physical changes on the hair surface were determined for the dyed hair sample. The best repair was seen when grape seed oil was applied to the hair. The dyed hair showed a higher roughness caused by the polymer deposition on the hair surface.

## 4 Conclusions

The study was carried out to observe the changes in hair because of herbal oil applications. It was observed that the most positive effect was achieved with grape seed oil, then rosehip oil and safflower seed oil. Shampoo had a lot of negative effects for hair samples. With the study, the damage caused to the hair by the raw materials in the shampoo was also evaluated. Herbal oils used were shown to reduce the debilitating effect of shampoo. The highest brightness value at 85 °C was read in the shampooed hair sample. The rupture elongation values of the reference hair were similar with the processed hair types between 27 and 62%. Also, gloss measurement values (L, *a*, and *b*) were obtained for all hair types in the range of 20–50; 3.9–12; and 3–20, respectively. Δ*E* values were not affected during the process and remained stable between 0.5 and 2.5. When all analyses were evaluated, it was seen that the best performing herbal oil was grape seed oil. The validation of all hair protocols was performed by repetitive trials at different crossover rates, considering the age range (25–55) and gender (male/female). Here, the application times and dosage amounts were also important. Particularly, in the study, trials were carried out by going through volunteers, considering the criteria given. In the light of the results obtained, the usability of herbal oils for different hair types was verified by deducing the appropriate protocols for the hair used in the experiments. As a result, it was concluded that this herbal oil could be widely evaluated for the cosmetic industry.
